# Gametogenesis and Auxospore Development in *Actinocyclus* (Bacillariophyta)

**DOI:** 10.1371/journal.pone.0041890

**Published:** 2012-08-01

**Authors:** Masahiko Idei, Keigo Osada, Shinya Sato, Kensuke Toyoda, Tamotsu Nagumo, David G. Mann

**Affiliations:** 1 Department of Biology, Bunkyo University, Saitama, Japan; 2 Department of Biology, The Nippon Dental University School of Life Dentistry at Niigata, Niigata, Japan; 3 Royal Botanic Garden Edinburgh, Edinburgh, United Kingdom; 4 Department of Biology, Keio University, Kanagawa, Japan; 5 Department of Biology, The Nippon Dental University, Tokyo, Japan; University of Melbourne, Australia

## Abstract

cGametogenesis and auxospore development have been studied in detail in surprisingly few centric diatoms. We studied the development of sperm, eggs and auxospores in *Actinocyclus* sp., a radially symmetrical freshwater diatom collected from Japan, using LM and electron microscopy of living cultures and thin sections. *Actinocyclus* represents a deep branch of the ‘radial centric’ diatoms and should therefore contribute useful insights into the evolution of sexual reproduction in diatoms. Spermatogenesis was examined by LM and SEM and involved the formation of two spermatogonia (sperm mother-cells) in each spermatogonangium through an equal mitotic division. The spermatogonia produced a reduced ‘lid’ valve, resembling a large flat scale with irregular radial thickenings. Sperm formation was merogenous, producing four sperm per spermatogonium, which were released by dehiscence of the ‘lid’ valve. The sperm were spindle-shaped with numerous surface globules and, as usual for diatoms, the single anterior flagellum bore mastigonemes. One egg cell was produced per oogonium. Immature eggs produced a thin layer of circular silica scales before fertilization, while the eggs were still contained within the oogonium. Sperm were attracted in large numbers to each egg and were apparently able to contact the egg surface via a gap formed between the long hypotheca and shorter epitheca of the oogonium and a small underlying hole in the scale-case. Auxospores expanded isodiametrically and many new scales were added to its envelope during expansion. Finally, new slightly-domed initial valves were produced at right angles to the oogonium axis, after a strong contraction of the cell away from the auxospore wall. At different stages, Golgi bodies were associated with chloroplasts or mitochondria, contrasting with the constancy of Golgi–ER–mitochondrion (G-ER-M) units in some other centric diatoms, which has been suggested to have phylogenetic significance. Electron-dense bodies in the vacuole of *Actinocyclus* are probably acidocalcisomes containing polyphosphate.

## Introduction

For many years the nature of sexual reproduction in centric diatoms was not understood. It was known that cell size was restored via auxospores, as in pennate diatoms, but it was thought that the auxospores were produced asexually from small vegetative cells. It was also known that the cells of some centric diatom species sometimes divided up to produce small naked cells, which were referred to as ‘microspores’ (e.g. [1], [2]), but these were not considered to be involved in auxosporulation. Following the discovery of meiosis during the formation of microspores in *Coscinodiscus* by Hofker [3] and observations of apparent attraction between small released flagellate cells and undivided cells of *Chaetoceros* by Went [4], Geitler [5] suggested that centric diatoms might be oogamous. However, it was not until 1950 that von Stosch [6] finally demonstrated oogamy, in *Melosira*. Since 1950, there have been many reports of spermatogenesis or auxospores in centric diatoms, leaving little doubt that oogamy is the only method of allogamous sexual reproduction in the group. However, most of these reports are of individual stages (e.g. in [7] or [8], [9]) and there are remarkably few centric diatoms in which the whole of the sexual process has been documented. The most complete reports are the series of five ‘Entwicklungsgeschichtliche Untersuchungen’ by von Stosch and colleagues on species of *Melosira*, *Odontella*, *Stephanopyxis* and *Chaetoceros* ([10], [11], [12], [13], [14]; see also [15]) and there is also a detailed early account of *Cyclotella* reproduction [16]. More recent papers deal with *Bacteriastrum* [17], *Attheya* [18], *Melosira* [19], [20], *Skeletonema* [21], *Coscinodiscus* [22], and *Thalassiosira* [23].

The centric diatoms that have been studied in detail represent several of the major lineages detected by molecular systematic studies (e.g. [24]). However, there are also lineages – such as *Hydrosera* and *Terpsinoë*, the *Toxarium* clade (*Toxarium*, *Climacosphenia* and *Ardissonea*), *Lampriscus*, *Leptocylindrus*, and the subject of the present paper, *Actinocyclus* – for which little or nothing is known of the sexual phase. It is ironic that Hasle et al. [25] excluded the Cymatosiraceae from the pennate diatoms largely because of their oogamy (which was clearly well-known to von Stosch, judging by his comments in [25] and [26]) and yet the details of this process and the resulting auxospores have never been published. Thus, much basic observation and description remains to be done before it will be possible to determine the evolution of the life cycle and sexuality in centric diatoms.

One of us (M.I.) discovered an undescribed freshwater *Actinocyclus* associated with sandy sediments in Lake Ogawara, Japan. This was isolated into clonal culture and its sexual reproduction studied by light microscopy (LM) of living and DAPI-stained cells, SEM observations of critical point dried material, and TEM of thin sections. The results of these studies are reported here, although a brief summary was given in [27]. Apart from this, the only information about sexual reproduction in *Actinocyclus* is a brief mention of merogenous sperm formation in *A. octonarius* by von Stosch (unpublished information in [13], p. 242, under *A. ehrenbergii*) and Subrahmanyan’s [28] short unillustrated report of microspore formation (spermatogenesis) in the same species. The structure of the vegetative cells of the Ogawara *Actinocyclus* and changes in valve morphology during the life cycle will be presented elsewhere, including a formal description of the new species as *Actinocyclus aquae-dulcis*, but it is treated as *Actinocyclus* sp. in this study.

Particular focuses of our study are spermatogenesis and oogenesis, and the scale case of the auxospore. In many centric diatoms, the auxospore is covered by a wall containing numerous siliceous scales, but it is unclear when scale formation begins. *A priori*, one might assume that, because plasmogamy requires cell membranes to touch and fuse, scales would be formed only after the egg has been fertilized by a sperm. We attempt to clarify whether this is so.

## Materials and Methods

*Actinocyclus* sp. was collected from Lake Ogawara (Ogawara-ko), Aomori Prefecture on 30 September 1989 and single cells were isolated by micropipette to establish clonal cultures, which were maintained in 1/5 BBM medium (modified Bold’s basal medium [20]) at 20°C and under 34.2–45.6 µmol m^–2^ s^–1^ provided by cool-white fluorescent tubes with a 12∶12 light-dark cycle. The original clone used for the ultrastructural analyses reported here has since died. Therefore, in order to provide evidence of the identity of the *Actinocyclus* species studied, new clones were established from single cells, both collected from Lake Ogawara on 12 June 2011 and Lake Akan (Akan-ko), Hokkaido on 28 May 2011. These were used to obtain *rbc*L sequences, in order to barcode the diatom in accordance with recent recommendations ([29], [30], [31]). In addition, the valve morphology of *Actinocyclus* sp. is documented in Figure S1. Cultures of *Actinocyclus* sp. are available from the first author upon request.

No change in culture conditions was required to induce spermatogenesis or oogenesis, which both occurred together in the same clonal cultures (i.e. the diatom is homothallic). For light microscopy of living cells, we used a Nikon Optiphoto light microscope with Nomarski optics. DAPI staining was performed with 1 µg ml^–1^ DAPI (4,6-diamino-2-phenylindole) in a modified S buffer [32]. For SEM, cells were fixed with 2% glutaraldehyde, 0.25 M sucrose, 0.2 M cacodylate buffer at room temperature. They washed with cacodylate buffer and dehydrated in a graded series of ethanol, and critical point dried using HITACHI HCP-2 equipment, coated with gold-palladium (Fine Coat JFC-1100) and observed using a JEOL-F15 field emission SEM at an accelerating voltage of 15 kV and tilted up to 45° angle. Ultrathin sections were prepared by fixing cells with 1% glutaraldehyde and 0.5% OsO_4_ prepared in the culture medium, dehydration in an ethanol series, and flat-embedding in Spurr’s resin [33]; vegetative cells, oogonial cells and auxospores were then selected using LM (choosing cells that appeared to typical of their stage, judging by LM observations of living cells), reorientated, photographed (Figure S2), sectioned using Leica Reichert Ultracut S, and examined using a JEOL JEM-1200EX.

For voucher *rbc*L sequences, DNA was extracted from two *Actinocyclus* sp. clones (A5 and D3) from Lake Akan and one (A6) from Lake Ogawara, using the method described in [34]. The *rbc*L sequences obtained were c. 1,480 bp long, representing most of the gene, and have been deposited in GenBank as accessions AB723750 (A-5), AB725385 (D-3) and AB725386 (A-6). Apart from one possible nucleotide difference between one of the Ogawara sequences and the other two, the sequences were identical.

## Results

Cells were approximately 40–48 µm in diameter when isolated and were immediately capable of forming eggs and sperm. It has not yet been possible to determine the sizes at which cells become capable of oogenesis or spermatogenesis, since F1 clones are still large-celled (>90 µm).

**Figure 1:**
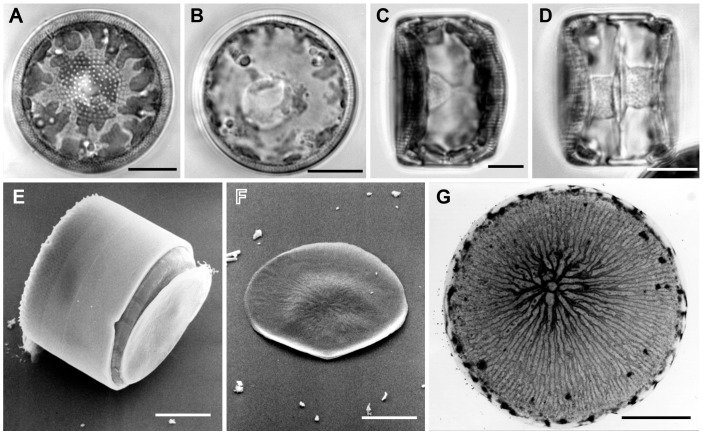
Vegetative cell and lid-like valve in *Actinocyclus.* A–D, LM. E, F, SEM. G, TEM. A–C. Vegetative cells. **A, B**. Valve view showing lobed chloroplasts and a central nucleus. **C**. Girdle view showing a nucleus and a thin peripheral layer of cytoplasm. **D**. Girdle view of two spermatogonia formed by an equal mitotic division. **E–G**. Lid-like valves. **E**. Spermatogonium with a lid-like valve. **F**. Detached lid-like valve. **G**. A lid-like valve with ribs radiating from an annulus. All scale bars = 10 µm, except G (5 µm).

Vegetative cells were drum-shaped (Figs. 1A–C) and c. 20–30 µm deep during interphase. They contained a ± central nucleus, suspended between the valves in a central column or pocket of cytoplasm (Figs. 1B, C), and a thin peripheral layer of cytoplasm containing lobed chloroplasts (Fig. 1A), which were distributed more or less evenly across the valves and girdle. Most of the cell lumen was occupied by a large vacuole (Figs. 1B, C).

### Spermatogenesis

The formation of sperm began with an equal mitotic cell division of the spermatogonangium (Fig. 1D), which up to that point did not differ visibly from vegetative cells. Following cell division, however, the daughter cells (spermatogonia) did not form normal loculate valves. Instead, each produced a flat disc (Figs. 1E, F) bearing a somewhat irregularly branched, radiating pattern of ribs, which were thicker towards the centre and subtended by an annulus (Fig. 1G). The disc had no rimoportulae, areolae or cingulum. The spermatogonia therefore appeared as modified cells, each with a normal theca inherited from the spermatogonangium and a lid-like disc-valve (Fig. 1E).

**Figure 2:**
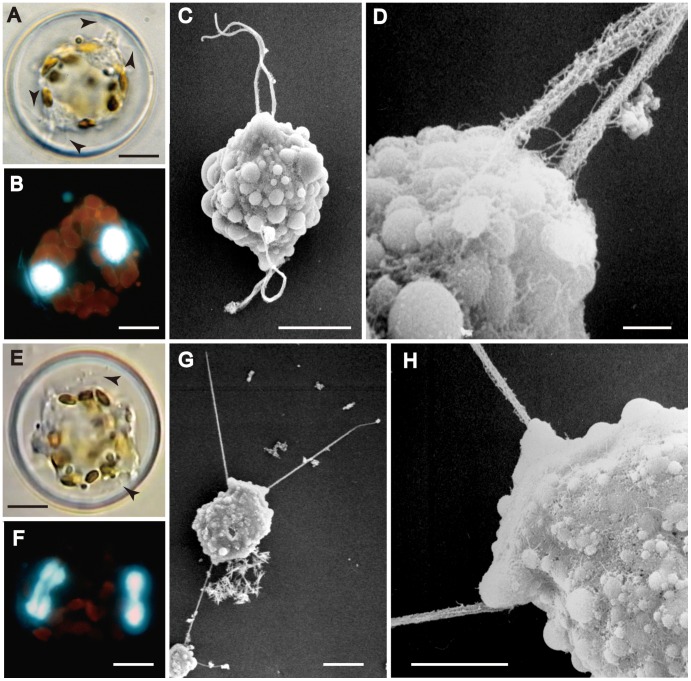
Spermatocyte after meiosis I in *Actinocyclus*. A, B, E, F, LM. Figs. C, D, G, H, SEM. A–D. **A**. Cell with paired flagella (arrows) associated with each nucleus. **B**. DAPI-stained cell with two separated nuclei. **C**. Spermatocyte with peripheral vesicles and elongating paired flagella on each side, which have reached about 1/3 of their final length. **D**. Enlargement of basal area of the paired flagella with mastigonemes. **E–H**. Spermatocyte during meiosis II. **E**. Cell undergoing nuclear division with separated flagella (arrows). **F**. DAPI-stained cell showing dividing nuclei (the median vertical line in the left-hand nucleus marks the position of the spindle). **G**. Cell with separated flagella on either side of two flat projections where the nuclei are located. **H**. Enlargement of a flat projection, with flagella extending out on either side. Scale bars = 10 µm except D (1 µm) and H (5 µm).

**Figure 3:**
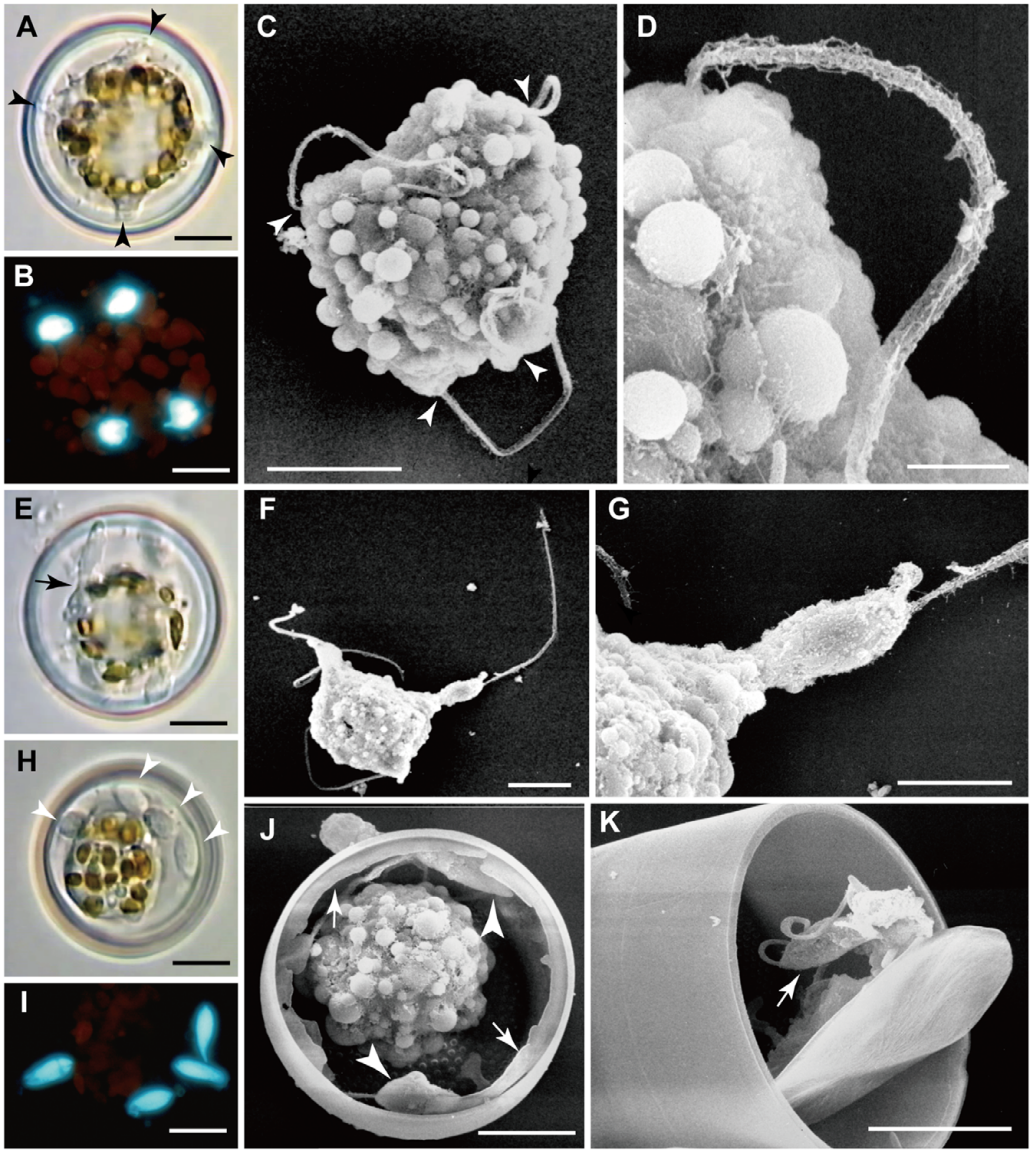
Spermatocyte after meiosis II in *Actinocyclus*. A, B, E–G, LM. Figs. C, D, H–K, SEM. A–D. **A**. Cell with four projections (arrows), each bearing a single flagellum. **B**. DAPI-stained cell with four separated nuclei. **C**. Cell with four flagella extending from four projections (arrows). **D**. Enlargement of a projection with a flagellum with mastigonemes. Note narrower posterior end. **E**. Final stage of spermatogenesis: sperms separating from the cell (e.g. at arrow). **F**. Sperm with almost fully elongated flagellum being pinched off from the cell. **G**. Enlargement of F. **H**. Four separated sperms (arrows) and a large residual body in a spermatogonium. **I**. DAPI-stained cell at a similar stage to H, with four sperms in a spermatogonium. **J**. Spermatogonium with a residual body and uniflagellate sperms (arrowheads) and an apparently fragmentary lid valve around the margin (arrows). **K**. A spermatogonium with a partly detached lid valve and sperm inside (arrow). All scale bars = 10 µm except Figs. D (2 µm) and G (5 µm).

In the cells observed, there were no further mitotic divisions during spermatogenesis. Instead, each spermatogonium became transformed into a primary spermatocyte and underwent meiosis (Fig. 2A). During or after meiosis I, the protoplast of the primary spermatocyte contracted away from the spermatogonium wall and two sets of paired flagella could be detected growing out, one on each side of the cell, immediately above one of the nuclei (Fig. 2A). Meiosis I took place parallel to the valves of the spermatogonium, so that DAPI staining revealed two well-separated nuclei lying near the periphery of the cell when the spermatogonium was seen in valve view (Fig. 2B). SEM observations of developing spermatocytes illustrate the formation of the flagella, and suggest a highly dynamic cell surface, with peripheral vesicles and likely active secretion (Figs. 2C, D). The flagella elongated and then, during the second meiotic division, which also took place in the same plane as the first division, separation of the two flagella of each pair began (Figs. 2E–H), Following the division, each flagellum was separate and had a nucleus associated with its basal part (Figs. 3A–D). Elongate sperm bodies began to be form on the surface of each beneath each flagellum, but for some time their ends remained attached to the spermatocyte (Figs. 3E–G). Finally, uniflagellate sperm were pinched off from the cell, leaving a large residual body containing the chloroplasts (Figs. 3H–J), which were now contracted and granular. Spermatogenesis was therefore merogenous. Sperm were released by dehiscence of the disc-valve of the spermatogonium (Fig. 3K), which was apparently facilitated by the vigorous movement of the sperm themselves.

**Figure 4:**
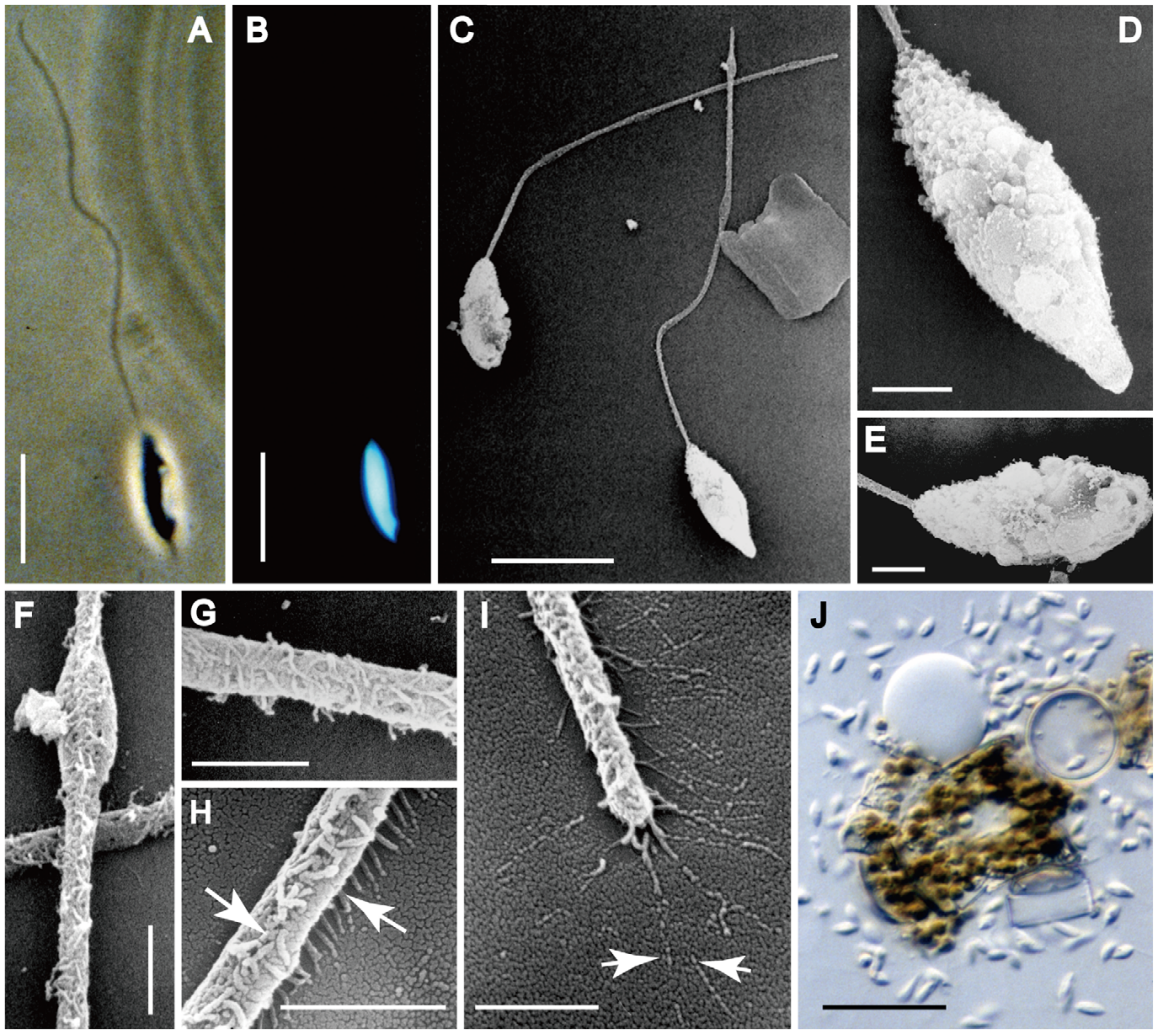
Sperm morphology and flagellar fine-structure in *Actinocyclus*. A, B, J, LM. C–I, SEM. **A, B**. Phase contrast and DAPI images of a sperm showing long anterior uniflagellate and an elongate nucleus occupying most of the sperm ‘body’. Scale bars = 10 µm. **C**. Two sperm: both are elongate but one is more pointed than the other. Note that one sperm bears a swelling near the tip of the flagellum. Scale bar = 10 µm. **D, E**. Enlargement of the sperm shown in Fig. 29. Scale bars = 2 µm. **F**. Enlargement of the swollen portion of one flagellum shown in Fig. 4C: note also the line of mastigonemes (pointing towards the viewer). Scale bar = 1 µm. **G**. Detail of mastigonemes. Scale bar = 1 µm. **H**. Detail of mastigonemes lined in a row at both sides of the flagellum. Scale bar = 1 µm. **I**. Detail of mastigonemes, showing that their tips (arrows) appear branched, because they bear terminal filaments (arrows). Scale bar = 1 µm. **J**. Many sperms crowding around a broken egg (center). Scale bar = 50 µm.

The body of each sperm (Fig. 4A) and the nucleus within it (Fig. 4B) were elongate (8–10 µm long and ca. 2.5–3 times as long as wide) and bore a long anterior flagellum (34–40 µm: Figs. 4A, C). The morphology of the sperm body varied slightly, since the end distal to the flagellum could be more or less acute to round (Figs. 4D, E, respectively). Sometimes small expansions or blobs of unknown nature were present along the flagellum (Figs. 4C, F). There were two lines of mastigonemes (Figs. 4G–I). Sperm were strongly attracted to any eggs present nearby and clustered close to it (even if the egg was killed by squashing: Fig. 4J). Fertilization was not observed directly, but no auxospore development took place unless sperm were present at the same time as egg cells.

### Oogenesis

**Figure 5:**
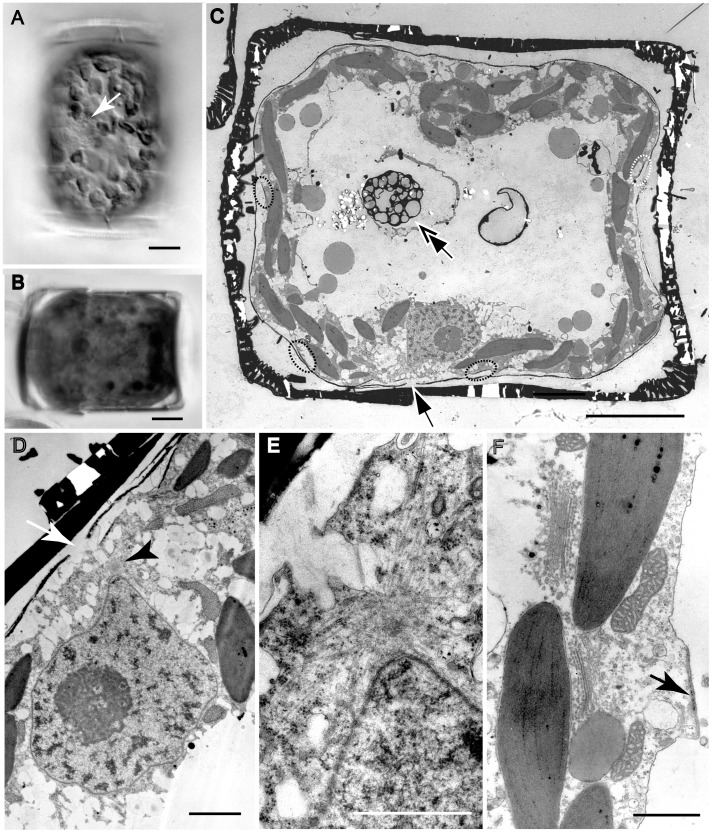
Early stage oogonial structure in *Actinocyclus*. A, B, LM. E–F, TEM. **A**. Peripheral focus of a fully elongated oogonium containing a single nucleus with a nucleolus (arrow), located just beneath the girdle. Scale bar = 10 µm. **B**. A young oogonium before thin sectioning: note the unequal epi- and hypothecae. Scale bar = 10 µm. **C**. The same oogonium as in Fig. 5B after thin sectioning. The cell is still fully enclosed by the hypotheca and epitheca. Note a thin layer of scales (appearing as a black line) surrounding the cytoplasm, several scales being formed beneath the cell membrane (circles), and a small discontinuity in the scale layer (arrow). Within the vacuole are several bodies containing ‘frothy’ electron-dense material (double arrows), as well as smaller grey bodies that probably represent lipid. Scale bar = 10 µm. **D**. Enlargement of **C** showing a pear-shaped nucleus (with a single nucleolus) elongated towards a centrosome (arrowhead) and overlying split in the scale case (white arrow). Scale bar = 2 µm. **E**. Detail of the centrosome located at the tip of the nucleus and subtending many radiating microtubules. Scale bar = 1 µm. **F**. A peripheral part of the cell showing Golgi bodies associated with chloroplasts; note also the mitochondria and a vesicle (arrow) containing a scale beneath the cell membrane. Scale bar = 1 µm.

**Figure 6:**
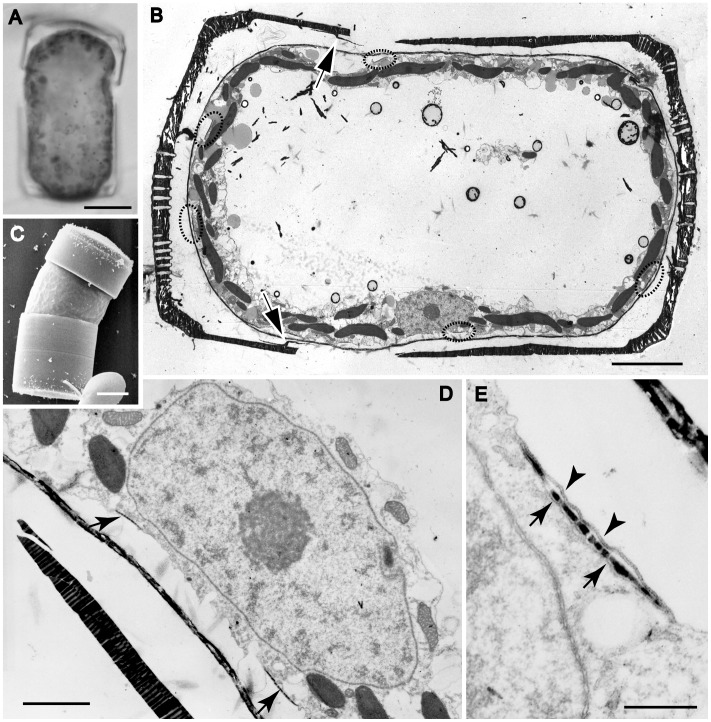
Middle stage oogonial structure in *Actinocyclus*. A, LM. B, D, E, TEM. C, SEM. **A, B**. The same elongated, dehisced oogonium, which has split apart within the hypotheca, leaving one of the hypothecal band attached to the epitheca. Scale bars = 10 µm. **B** shows a complete layer of scales around the cell, scale forming vesicles (circles), and a nucleus located at the side, beneath the girdle of the oogonium. Note the distal band attached to the inside of the epivalve (arrows). Scale bars, 10 µm. **C**. An oogonium at ± the same developmental stage as **A**, showing a deeper hypotheca than epitheca (towards the top, lacking girdle bands) and an exposed cell covered by a siliceous layer visible between the thecae. Scale bar = 10 µm. **D**. Detail of the nucleus in the cell shown in Fig. 6B, showing the broken edge of the hypotheca (left), the layer of scales (two scales thick), scale-forming vesicles (arrows), and mitochondria and chloroplasts (but no Golgi bodies) surrounding the nucleus. Scale bar = 2 µm. **E**. Detail of scale silicadepositing vesicle (SDV), showing two membranes externally (arrowheads), representing the plasma membrane and the outer membrane of thescale SDV, and a single one internally (arrows). Note numerous closely-spaced pores in the nuclear envelope and no associated Golgi bodies. Scale bar = 0.5 µm.

**Figure 7:**
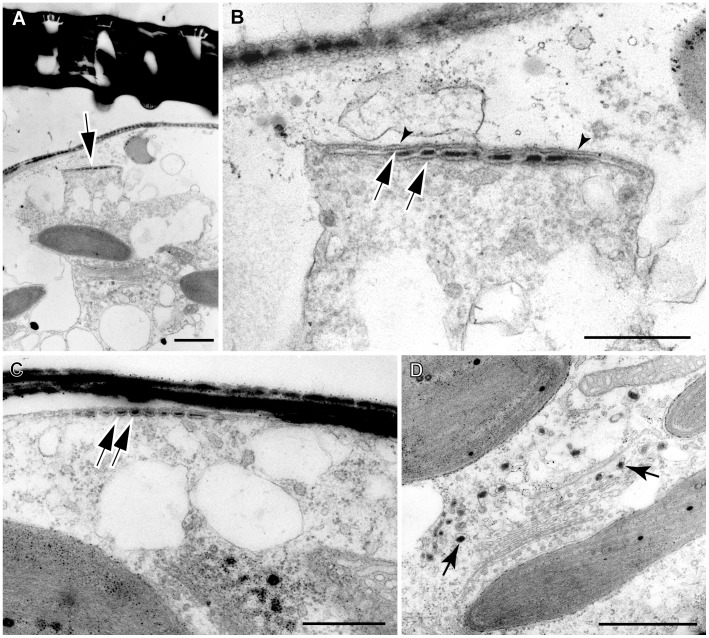
Details of scale SDV in *Actinocyclus*. A–D, TEM. **A**. A part of oogonium showing a Golgi body associated with a chloroplast, and a scale SDV slightly separated from a layer of scales (arrow). Scale bar = 1 µm. **B**. Enlargement of the scale SDV of Fig. 7A, showing the SDV membranes (arrows) and the plasma membrane (arrowheads), which in this case has separated from the oogonium wall, probably as an artifact of specimen preparation. Note that the SDV membranes here are continuous but the silica within discontinuous. Scale bar = 0.5 µm. **C**. Detail of scale SDV (arrows), sectioned tangentially near its periphery, showing individual vesicle-like membrane profiles containing silica: these represent finger-like extensions of the SDV before fusion. Scale bar = 1 µm. **D**. Detail of a Golgi body associated with a chloroplast (therefore with chloroplast ER) and apparently producing many vesicles containing dark content (e.g. arrows). Scale bar = 1 µm.

**Figure 8:**
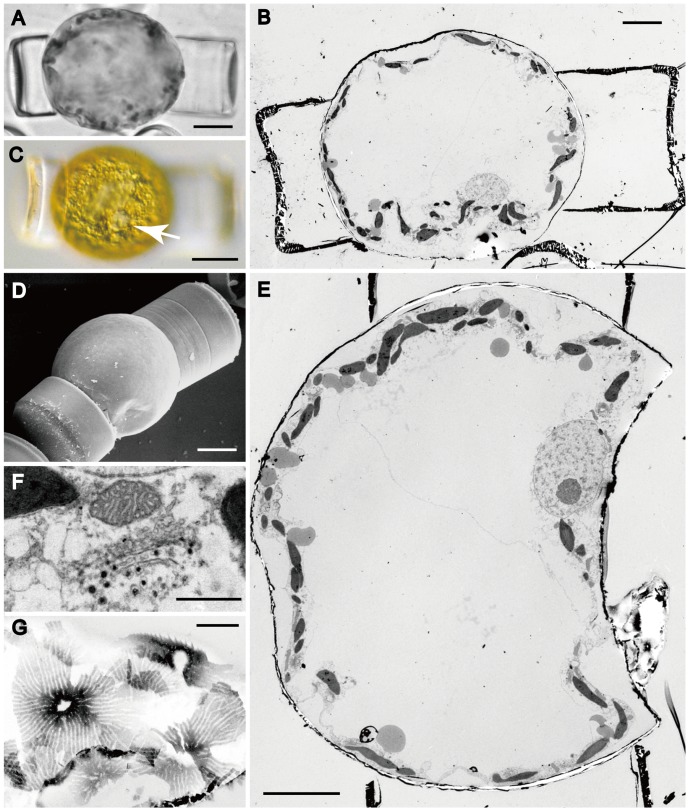
Young auxospore in *Actinocyclus*. A, C, LM. B, E–G, TEM. D, SEM. **A**. A young, partly expanded auxospore, still associated with the oogonium hypotheca and epitheca. Scale bar = 20 µm. **B**. A thin section of the auxospore illustrated in **A**, showing organelles arranged peripherally and a large central vacuole. Scale bar = 10 µm. **C**. A young auxospore showing peripheral chloroplasts and a single nucleus (arrow). Scale bar = 20 µm. **D**. A young auxospore completely covered by a layer of scales. Scale bar = 20 µm. **E**. Another thin section of the auxospore shown in Fig. 8A, passing through the centre of nucleus. Note that the nucleus is bigger than that of oogonium. Scale bar = 10 µm. **F**. Close association of a mitochondrion and a Golgi body producing vesicles with electron-dense contents. Scale bar = 1 µm. **G**. Scales with a central annulus and branching radial ribs. Scale bar = 1 µm.

Unlike spermatogonangia, which had more or less the same dimensions as vegetative cells, cells differentiating as oogonia (Figs. 5, 6 and 7) became noticeably elongated along the pervalvar axis, c. 65 µm rather than c. 35 µm (Figs. 5A, B, compare Fig. 1C). This was caused by modification of the girdle, the cingulum of the hypotheca being deeper than usual (Figs. 5B, C, 6A–C) and containing five or six bands (Fig. 8D; perhaps four in some cases) rather than the three present in vegetative cells. Curiously, however, none of the oogonia observed in LM or in thin sections possessed an epicingulum: the epitheca comprised only a valve (Figs. 5C, 6B, C, 8A–D); the girdle band seen attached to the epivalve in dehisced oogonia is in fact the pleura of the hypotheca, as explained later.

The nucleus was at first central within the oogonium, as in vegetative cells, and was suspended in the vacuole by cytoplasmic strands. Later, it moved to the one side of the cell (Fig. 5C), and thin sections showed that it was preceded by a small dense centrosome (microtubule organizing centre) subtending many radiating microtubules (Figs. 5D, 6A). At this stage the nucleus was pear-shaped (Fig. 5D) and appeared to be drawn by the centrosome, with microtubules running along the surface of the nuclear envelope (Fig. 5E). The cytoplasm was almost entirely peripheral and packed with chloroplasts and mitochondria (Fig. 5C); it was also highly vesiculate, both towards the central vacuole and in a narrow layer between the chloroplasts and the cell membrane. Golgi bodies (dictyosomes) were scattered through the cytoplasm, most or all being closely associated with a chloroplast via its forming face (Fig. 5F); there was no ‘shell’ of Golgi bodies around the nucleus (Fig. 5D). The distribution of Golgi bodies was also checked in two vegetative cells and here too they were associated with chloroplasts via their forming face (not illustrated). Lipid droplets were common in both the cytoplasm and the vacuole, and the vacuole also contained one or more bodies with electron-dense but ‘frothy’ content (Fig. 5C double arrows).

Fertilization was not observed but we observed several elongated oogonia in which the oogonial wall had split apart, partly exposing the egg within (Figs. 6A–C). We expected that this had occurred simply through separation of the hypotheca and epitheca of the oogonium. Careful examination revealed, however, that the split had in fact taken place within the hypotheca, leaving the distal (most abvalvar) band attached to the inside of the epivalve (Fig. 6B arrows). Dehiscence was slightly unequal, so that the egg was more exposed on the side where the nucleus lay (Fig. 6B).

Even more surprising than the splitting of the hypotheca was that the egg cell secreted a thin layer of scales before its final elongation and before the dehiscence of the oogonium wall to allow fertilization. This was true in all three of the young unexpanded oogonia that were sectioned (Fig. S2). Fig. 5C shows one of these young oogonia before elongation, which had already acquired an almost complete covering of scales. In this cell the cell membrane was naked immediately above the centrosome and nucleus (Fig. 5D arrow), which may indicate that an area of the cell surface is left uncovered as a fertilization pore. However, since we observed this in only one of the young oogonia, we cannot confirm whether the absence of scales above the nucleus, forming a ‘fertilization pore’, is a general feature. In the dehisced oogonium shown in Fig. 6B, the scale layer is continuous above the nucleus; possibly this cell had already been fertilized, with the sperm nucleus outside the plane of the section.

**Figure 9:**
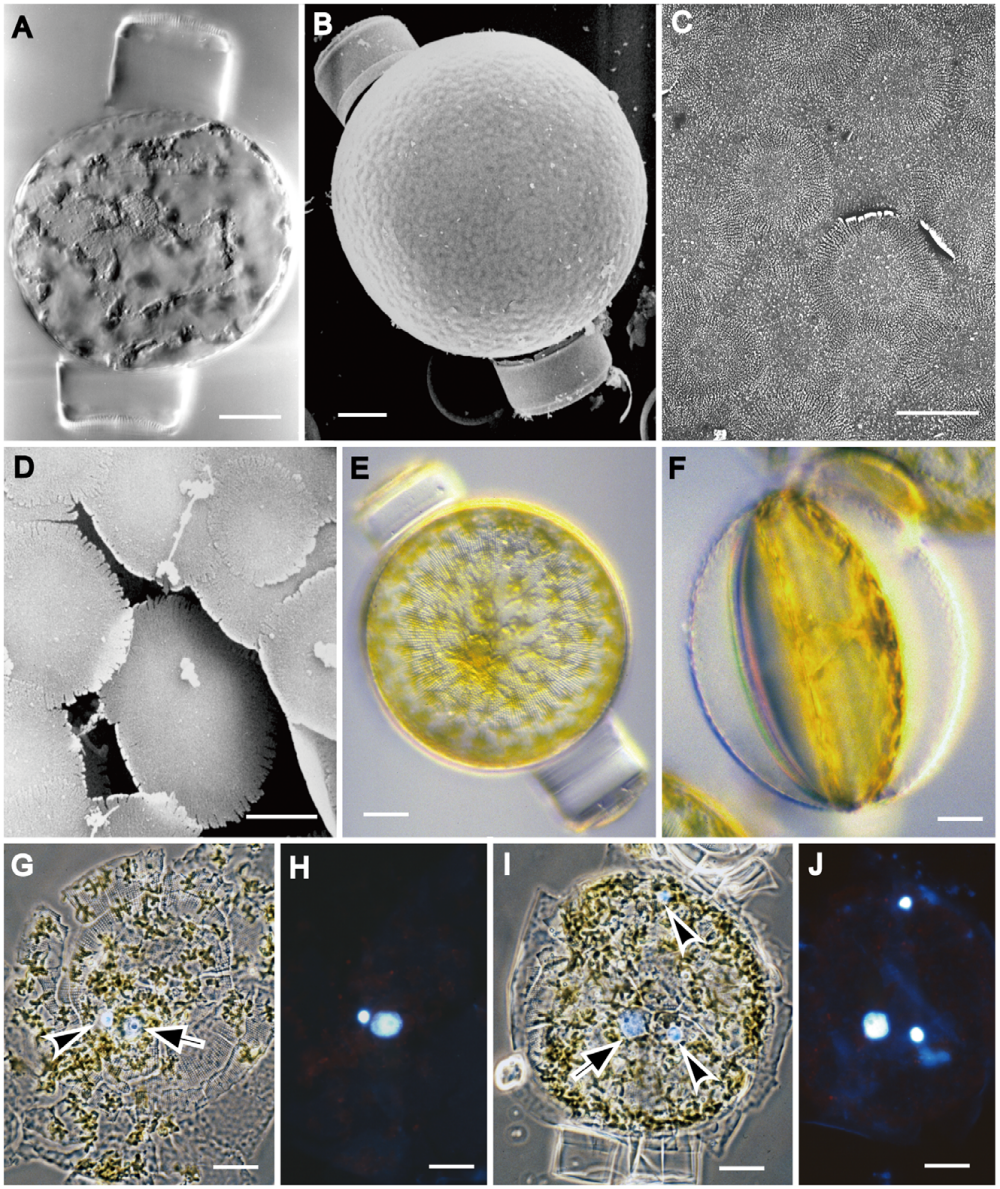
Fully expanded auxospore in *Actinocyclus*. A , E–J, LM. B–E. SEM. **A**. A spherical auxospore expanded between the hypotheca (top) and epitheca of the oogonium, with peripheral chloroplasts and a nucleus. **B**. Fully expanded auxospore covered by numerous scales. **C**. Enlargement of scales, each with a hyaline centre and fimbriate margin (cf. Fig. 9A). **D**. Broken auxospore wall showing overlap of scales differing in size. **E**. Initial cell in valve view, still enclosed within the auxospore and containing many stellate chloroplasts and a nucleus near its centre. **F**. Girdle view of a fully formed initial cell enclosed within the auxospore, showing the ± spherical auxospore wall and the rugby ball–like profile of the initial cell, formed as a result of two lateral contractions of the protoplast. **G**, **H**. DAPI-stained auxospore with initial epivalve, showing a nucleus (arrow) and a pyknotic nucleus (arrowhead in Fig. 9G). **I**. **J**. DAPI-stained auxospore with both initial epivalve and initial hypovalve, showing a nucleus (arrow) and two pyknotic nuclei (arrowheads in Fig. 9I). the slightly smaller pyknotic nucleus (top) derived from the acytokinetic mitosis preceding formation of the epivalve, the other from the equivalent mitosis preceding formation of the hypovalve. Scale bars = 20 µm. except C, D (2 µm).

Scales were formed in the peripheral cytoplasm, immediately below the cell membrane (Figs. 6D, E, 7A, C), and then released through exocytosis. As with other silica structures in diatoms, each scale was produced in its own flattened silicon deposition vesicle (SDV). Tangential sections through forming scales (Fig. 7C) revealed that the branching ribs of each scale are formed in finger-like extensions of the SDV, so that the relationship of the ribs to the SDV is as fingers in a glove, rather than fingers in a mitten (this analogy is due to [35]). In developing oogonia and auxospores, vesicles with dense ontent, apparently condensed silica, were observed both within the cytoplasm and budding off from Golgi bodies (Figs. 5F, 7D). There was no evidence that scales were moulded on the surface of chloroplasts or other organelles, nor that scale formation was restricted to particular parts of the surface of the egg cell. All scales were circular or slightly elliptical, with faint radial markings and consisted of a central ring (annulus) surrounded by a fringe of delicate branching ribs (Figs. 8G, 9C, D).

After presumed fertilization, the egg (zygote) contracted away from the oogonium wall, becoming ellipsoidal and then ± spherical, and then began to expand isometrically as an auxospore (Figs. 8A–E, 9A, B). During expansion, more scales (apparently identical in structure to those formed earlier but perhaps larger on average: Fig. 9D) were added to the auxospore wall, so that the auxospore remained completely covered by scales (Fig. 9B), with approximately constant wall thickness (Figs. 6B, 8E). Throughout expansion and during the formation of the initial valves, the thecae of the oogonium remained attached to opposite sides of the auxospore (Figs. 8A–D, 9A, B, E), making the same slight angle to each other as they did following dehiscence (Figs. 6C, 8D, 9B). In expanding auxospores, the nucleus was noticeably larger than that in the oogonium (compare Fig. 8E with Figs. 5C and 6B), suggesting it was now diploid. It remained at the periphery of the cell, near the centre on one side (Figs. 8C, E). In auxospores, we observed examples of Golgi bodies associated with mitochondria, rather than with chloroplasts (Fig. 8F). The centre of expanding auxospores was occupied by a large vacuole (Figs. 8B, E).

Each initial valve was formed after a unilateral contraction of the protoplast, so that its margin was the only part moulded by the auxospore wall (Fig. 9F). The initial cell was orientated at right angles to the oogonium and contained many stellate chloroplasts (Fig. 9E), both in the thin peripheral layer of cytoplasm and in cytoplasmic strands radiating from the nucleus. Prior to the formation of each initial valve there was an acytokinetic mitosis, after which the superfluous nucleus degenerated (Figs. 9G–J).

## Discussion

### Evolutionary Implications

A close association between three organelles – a Golgi body, endoplasmic reticulum and a mitochondrion – has been reported in two species of *Coscinodiscus* [36], *Ellerbeckia* [37], *Stephanopyxis* (Wurzinger & Schmid, personal communication to [38] and see [36]), and in *Odontella* [39]. Schmid [36] noted that, among other heterokonts, G-ER-M units (terminology of [36]) have been found in the xanthophyte *Vaucheria* (e.g. [40]) and the oomycete *Saprolegnia* [41]. The single Golgi body of bolidophytes (the closest relative of diatoms) is also accompanied by a mitochondrion [42], but bolidophytes have such small cells that all organelles are in close proximity. So far, no Golgi apparatus has been demonstrated in parmophytes, even though these and bolidophytes seem to represent different stages in the life cycles of the same group of organisms, given the nested phylogenetic position of parmophytes in the robust bolidophytes clade [43]. The occurrence of G-ER-M units in several heterokonts outside the diatoms and also in several diatom lineages that apparently branched from each other early during diatom evolution could suggest that the G-ER-M arrangement is ancestral in diatoms, as suggested by Medlin et al. ([38]: see their fig. 1).

According to the 18 S rDNA, *rbc*L+*psb*C, and combined gene trees presented by Theriot et al. [24], *Actinocyclus* represents a different deep branch within the diatoms to the branches leading to *Coscinodiscus* and *Stephanopyxis*, which are both G-ER-M taxa, and it might be expected that, if the G-ER-M arrangement has the phylogenetic significance ascribed to it by Medlin et al. [38], *Actinocyclus* would also possess G-ER-M complexes. However, in *Actinocyclus*, the association between Golgi bodies and other organelles seems to be flexible. Sometimes there is an association between Golgi bodies, not with mitochondria, but with chloroplasts and hence with the ‘chloroplast endoplasmic reticulum’ (CER) that bounds the chloroplasts in the four-membrane-bound chloroplasts of diatoms and other heterokonts; sometimes there does appear to be an association with mitochondria, but not with ER or CER. Quite possibly, then, the associations of the Golgi bodies change during the life-cycle or cell-cycle of *Actinocyclus*. Furthermore, both the G-ER-M arrangement and the other common arrangement of Golgi bodies in diatoms – in a shell around the nucleus (references in [44] and see [45]) – are found in different species of *Odontella*: see [39], p. 217). The evolutionary significance of G-ER-M units is therefore less clear than has sometimes been suggested [38], [1]. Clearly, if organelle associations are to be used to build or check phylogenetic hypotheses, it is important to make comparisons between cells at equivalent stages of their cell or life cycles. Direct comparison is possible between the vegetative cells of *Actinocyclus* and *Coscinodiscus* and these show a clear difference, those of *Actinocyclus* having Golgi–chloroplast associations, whereas *Coscinodiscus* has G-ER-M [36]. Furthermore, we have found no cell of *Actinocyclus* (no vegetative cell or auxospore, nor any stage in oogenesis) in which G-ER-M units are present, which leaves spermatogenesis as the only stage for which there is any doubt about the Golgi associations in this diatom.

Medlin & Kaczmarska ([46], p. 262) also reviewed spermatogenesis in their search for morphological, developmental and cytological features that might be synapomorphies of molecular clades (principally from analyses of rDNA). They suggested that the shape of the sperm nucleus and the process of sperm formation might have phylogenetic significance, elongate sperm nuclei and merogenous sperm formation being thought to be characteristic of radial centrics (Medlin & Kaczmarska’s ‘clade 1’, which is not monophyletic in many gene trees (e.g. [24]). Sims et al. ([2], p. 394) were more cautious, noting that centric diatom lineages needed to be much better sampled for reproductive characters before patterns could be detected in the evolution of spermatogenesis and oogenesis. Thus, in opposition to Medlin & Kaczmarska’s suggestion that the characteristics of spermatogenesis largely support separation of radial centrics (merogenous) from polar centrics and pennates (hologenous), Jensen et al. [3] and Mizuno [4] have tabulated data indicating that hologenous and merogenous sperm formation can even occur in different species of the same genus, with little obvious pattern in their distribution among families and orders. Nevertheless, *Actinocyclus*, which belongs among the radial centric lineages according to multi-gene phylogenies [24], exhibits merogeny (see also [28], [13]) and has elongated sperm nuclei, in accordance with Medlin & Kaczmarska’s generalization about radial centrics.

### Cell Ultrastructure

Most of the ultrastructural features revealed here, such as the presence of a centrosome (microtubule organizing centre) and its role in mediating nuclear movements, the structure of the chloroplasts and mitochondria, formation of silica structures within SDVs, etc., are well-known for diatoms (e.g. [46], [47]). However, a few features are worth emphasis. We observed small Golgi-derived vesicles with dark contents, similar in electron-opacity to silica, in cells (oogonia and auxospores) that were actively engaged in producing siliceous scales. Similar vesicles have long been observed in diatoms (e.g. [35]) and have sometimes been referred to as ‘silica [or silicon] transport vesicles’ (STVs) because of a possible role in transporting silicon to the SDV. However, although there often appears to be a good correlation between the production of dark-vesicles and silicification (e.g. [35], [48]), extended by our observations here, the role of the ‘STVs’ remains unclear, since it has never been proved that the dark contents are siliceous (e.g. [52], [49]).

The electron-dense bodies with ‘frothy’ content observed within the vacuoles of *Actinocyclus* oogonia (Fig. 5C) resemble the electron-dense bodies found in the vacuoles of *Gomphonema* and demonstrated to be polyphosphate [50]. Similar bodies occur in *Surirella* [51] and correspond to the ‘volutin’ granules described long ago by Heinzerling [52]. Volutin has been determined to be polyphosphate in various organisms and seems often to be part of a relatively recently recognized organelle, the acidocalcisome (see [53], [54], [55]), thought to function in storage (of metal ions, P, basic amino acids, and energy), osmoregulation, and pH homeostasis. We suggest that the ‘volutin granules’ of diatoms deserve further study, especially in view of the important geochemical role of polyphosphate that has been suggested (e.g. [56]).

The formation of each rib of the scales within a separate finger-like projection of the SDV follows the same pattern as rib formation in valves [50] and points to a uniform morphogenetic machinery, in which cells ‘measure’ across the cytoplasm or the cell membrane, rather than within the SDV itself. No current morphogenetic models seem to take this into account.

### Sexual Reproduction

Stages in spermatogenesis and auxospores are fairly often recorded in centric diatoms, but the critical phase of oogamous sexual reproduction – the fusion of a sperm with an egg cell – is usually assumed, not seen. If auxospores are formed only when both eggs and sperm are present, the assumption seems reasonable, but examples are known where auxospore formation is autogamous despite the simultaneous production of sperm (in *Cyclotella meneghiniana*, *Melosira nummuloides* and *Actinoptychus undulatus*: [57], [58]). In our cultures, auxospores were formed only when sperm were present, sperm were strongly attracted to oogonia, and oogonia elongated and bent to create an aperture through which sperm could gain access to the egg cell. These all suggest that *Actinocyclus* sp. is oogamous. The larger size of the auxospore nucleus, relative to that of the oogonium, also suggests that fertilization took place.

*Actinocyclus* sp. produces sperm within modified cells – the spermatogonia – which inherit a normal theca from the spermatogonangium and form a highly modified ‘lid’ valve, which appears to protect the spermatocytes and sperm until the sperm are mature. Then the lid breaks off (presumably through degradation of organic material linking it to the rest of the spermatogonium wall) and the sperm swim out. A residual body is left behind, containing plastids and cytoplasm. The lid valve is highly reduced, having a simple laminate structure and radiating ribs, rather than the complex ‘hypocaust’ and deep areolae of normal vegetative valves. It also lacks rimoportulae and girdle bands. In many respects, therefore, the lid valve resembles a scale – a larger version of the scales that surround the oogonium and auxospore – rather than a valve. However, its position (beneath the cleavage furrow of the divided spermatogonangium) and its formation following a mitosis (cf. [59]) both support interpretation as a valve. Reduced valves are known to be produced during spermatogenesis in other centric diatoms (e.g. in *Odontella*: [1] and [28], in both cases as *Biddulphia*), although their structure has rarely been studied in EM. Von Stosch et al. [14] illustrated reduced valves formed during spermatogenesis in *Chaetoceros*, which lacked the rimoportulae, setae and girdle of normal thecae. On the other hand, in *Ditylum* a rimoportule is apparently present, since a central spine, albeit reduced, can be seen in LM [60].

It is axiomatic that the anterior flagellum of diatom sperm bears tripartite mastigonemes (e.g. [51]), in accordance with the phylogenetic position of diatoms within the heterokonts, but it is surprising how few diatoms have in fact been checked for this character. The known examples are drawn from just four genera: *Chaetoceros* ([12], [14], [44]), *Actinoptychus* ([12], but without illustration), *Lithodesmium* ([61]), *Pleurosira* ([62], as *Biddulphia*). To these, we can now add *Actinocyclus*.

Oogenesis reveals a few novel features, particularly the lack of girdle bands in the epitheca and the dehiscence of the frustule between bands of the hypotheca rather than between epitheca and hypotheca.

### The Scale Case

It has been known for a long time (e.g. [63], [13]) that the auxospores of centric diatoms often bear small scales and this was one of the principal pieces of evidence used by Round & Crawford [64] to support their suggestion that the ancestors of diatoms were scaly unicells. Auxospore scales are usually circular or elliptical and flat, though in *Odontella* and Cymatosiraceae the outermost scales are 3-dimensional tree-like structures [26]. It has always been assumed that scales are formed only after fertilization, and indeed, it is very difficult to see how fertilization could occur if an egg was surrounded by several layers of robust scales (such as those surrounding the auxospores of *Ellerbeckia*: [33]) or by a thicket of spines (as in *Odontella aurita*: see fig. 11c in [26]). The formation of scales by the immature egg cells of *Actinocyclus* sp., albeit in a thin layer, is therefore surprising and a first such record for centric diatoms: scales are formed before elongation of the oogonium and hence before the egg cell is exposed and available for fertilization. Kaczmarska et al. [65], [66] presented evidence for scales on the gametes of *Pseudo-nitzschia* and *Nitzschia* species, and here too the presence of scales might be expected to hinder plasmogamy (see also [9], p. 108). The difficulty in such cases is knowing whether what has been observed is pathological (production of scales by gametes that would never have fused) or part of normal development. In *Neidium* a tight-fitting envelope (incunabula) of silica plates is usually formed around the young auxospore, but in some cases the plates are formed around incompletely fused gametes, or even around unfused gametes (DGM, unpublished observations), suggesting that, after gametogenesis, cells may sometimes be programmed to form scales, whether or not fertilization takes place. The enigma of scaly gametes can only be finally resolved by studying scale formation *in vivo* in cells that undergo fertilization and develop into auxospores; for this the PDMPO (2-(4-pyridyl)-5{[4-(2-dimethylaminoethyl-aminocarbamoyl)-methoxy]phenyl}oxazole) labeling technique could be used, in which the dye is co-deposited with silica into the newly synthesized siliceous structure [67] and we anticipate applying this method when suitable material is available in future. However, the observation of scales in all three immature oogonia of *Actinocyclus* sectioned, from healthy cultures that had been fixed rapidly for TEM, suggests that in this diatom scales are part of normal pre-fertilization development of the egg. Assuming that this is so, we speculate that scale formation could be an adaptation to growth in freshwater, functioning in osmoregulation during the exposure of the egg cell for fertilization, or immediately after fertilization to allow rapid closure of the fertilization pore apparently present in Figs. 5C and D. This hypothesis predicts that marine *Actinocyclus* (most *Actinocyclus* species are marine) will form scales only after fertilization. There appears to be no organic wall outside the scale layer, in contrast to *Melosira* [68].

Although *Actinocyclus* species possess a ring of large rimoportulae on the valve mantle [51] as in *Coscinodiscus*, we found no evidence of ‘slit scales’, which occur together with simple scales in the auxospore walls of *Coscinodiscus* and appear to bear a single rudimentary rimoportula (the ‘slit’) within the scale annulus [69]. Instead, all of the auxospore scales of *Actinocyclus sp.* are simple, round and flat, with a radial structure and fimbriate margins, like the auxospore scales of e.g. *Melosira* [67], [68], *Ellerbeckia* [33] or *Thalassiosira* [70]. The scales are flimsy and there are only two or three layers of them around the egg cell or auxospore, no matter what the stage of development. Their formation at the surface of the cell, in a thin layer of cytoplasm external to the plastids and containing mitochondria, is similar to *Cyclotella* [71].

### Initial Cell Orientation

Schmid & Crawford [33] made an interesting observation concerning the orientation of the initial cells relative to the oogonia in centric diatoms, noting that they often lie at a taxon-specific angle to each other, rather than being strictly parallel (as in the summary diagram of [62], fig. 9.5 for *Coscinodiscus* and related forms). Examples include *Pleurosira* ([72], as *Cerataulus*) and *Ellerbeckia* [33], where the axes of initial cells and mother cells lie at an oblique angle. In *Actinocyclus sp.*, the axes are ± perpendicular to each other, unlike some other diatoms with intercalary auxospores (auxospores that are accompanied on either side by the oogonial thecae), such as *Detonula pumila* or *Thalassiosira eccentrica* [8], where the axes are parallel. The functional and phylogenetic significance of these changes in orientation is unknown.

## Supporting Information

Figure S1 **Valve morphology in** ***Actinocyclus*** **.** A–D. Light micrographs. E–M. Scanning electron micrographs. **A–D.** Valves. Scale bars = 10 µm. **E.** External oblique girdle view of a whole frustule composed of concave and convex valves, wide valvocopula and pleura. Scale bar = 10 µm. **F.** External view of valve showing pseudonodulus (arrow) and external openings of labiate processes at valve margin (appearing as simple holes). Scale bar = 5 µm. **G.** External view of pseudonodulus. Scale, 1 µm. H. External view of areolae containing cribra, radiating from a central annulus. Scale bar = 2 µm. **I.** Internal view of valve showing fasciculate areolation and labiate processes. Scale bar = 10 µm. **J.** Internal view of valve mantle showing curved labiate processes and closer areolation. Scale = 2 µm. Fig. K. Internal oblique view of theca with wide valvocopula. Scale = 10 µm. Fig. L. External oblique view of broken end of valve, showing thick wall and tubular loculate areolae occluded by cribra at both surfaces. Scale = 1 µm. Fig. M. External oblique view of valvocopula with on fimbriate margin to the advalvar edge. Scale = 10 µm.
(TIF)

Figure S2 **Cells selected for sectioning** **in** ***Actinocyclus*** **.** A, C, E, G, I, K, L, N, P. Transmission electron micrographs. B, D, F, J, M, O. Light micrographs. B and C, D and E, F and G, H and I, J and K, M and N, O and P show the same cells respectively before and after thin sectioning. All scales = 10 µm. **A.** Vegetative cell with a central nucleus. **B, C.** Example 1 of a young oogonium with a thin layer of scales (visible as a thin dark line surrounding the protoplast (contrast S2-A), still fully enclosed by its frustule. **D, E.** Example 2 of a young oogonium with a thin layer of scales. **F, G.** Example 3 of a young oogonium with a thin layer of scales; in this cell, although the protoplast is still fully enclosed, the tilt of the epitheca (at top) relative to the hypotheca suggests that the oogonium was beginning to open to permit fertilization. **H, I.** Oogonium with slightly contracted cell after presumed fertilization. **J, K.** An expanded oogonium with a thin layer of scales. **L.** An ellipsoidal zygote/young auxospore with a thick layer of scales (contrast the thickness of the scale layer with e.g. S2-C, E, I or N), indicating new scale addition since presumed fertilization. **M, N.** A fully expanded oogonium after presumed fertilization, still with a thin layer of scales. **O, P.** An inflated, subspherical auxospore with a layer of scales.
(TIF)
