# Supplementary figures and images for: Gametogenesis and Auxospore Development in Actinocyclus (Bacillariophyta)
Source: PLoS One. 2012 Aug 1;7(8):e41890. doi: 10.1371/journal.pone.0041890 (PMC3411613; doi:10.1371/journal.pone.0041890)

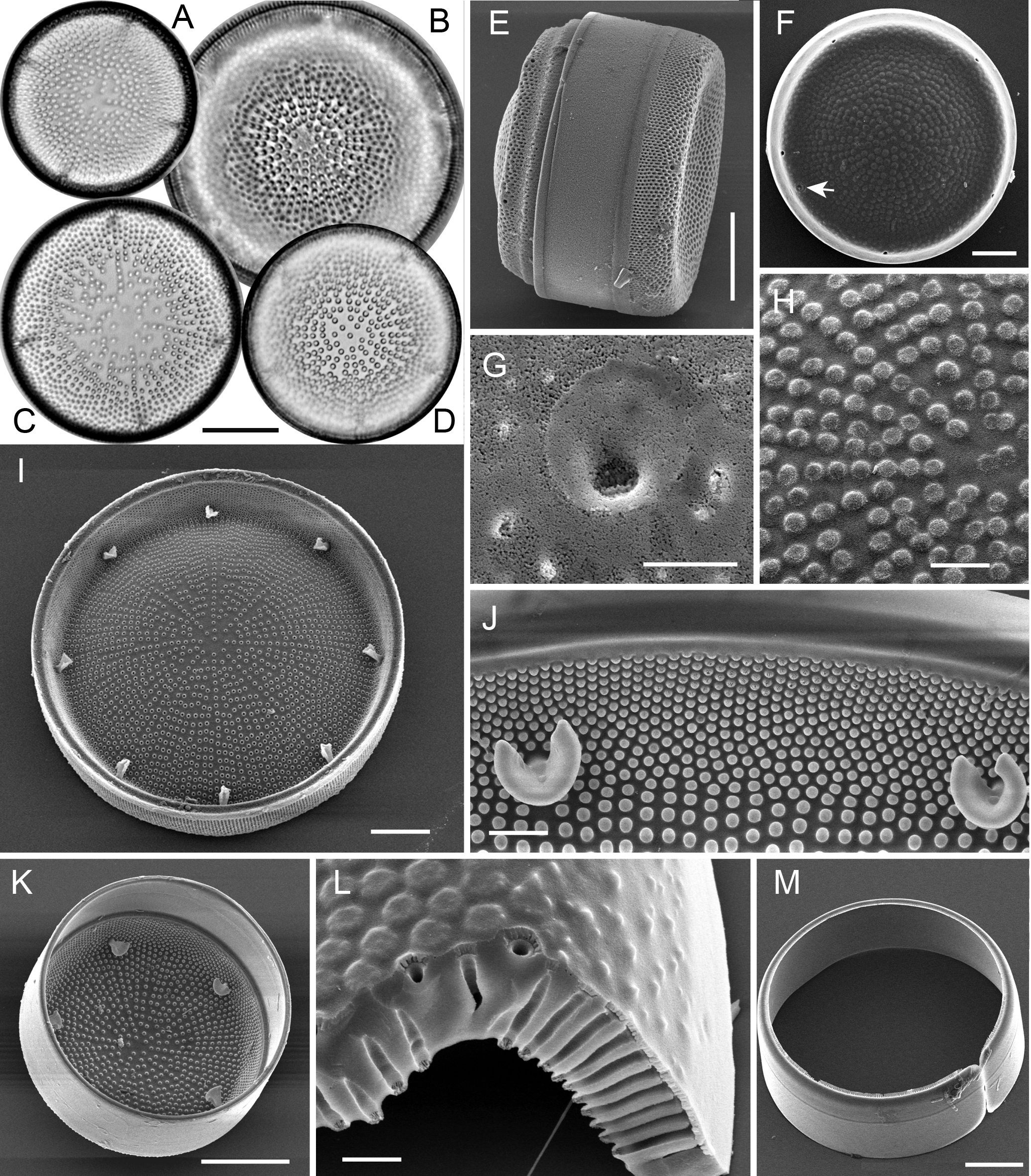

Supplement: Figure S1 — Valve morphology in Actinocyclus . A–D. Light micrographs. E–M. Scanning electron micrographs. A–D. Valves. Scale bars = 10 µm. E. External oblique girdle view of a whole frustule composed of concave and convex valves, wide valvocopula and pleura. Scale bar = 10 µm. F. External view of valve showing pseudonodulus (arrow) and external openings of labiate processes at valve margin (appearing as simple holes). Scale bar = 5 µm. G. External view of pseudonodulus. Scale, 1 µm. H. External view of areolae containing cribra, radiating from a central annulus. Scale bar = 2 µm. I. Internal view of valve showing fasciculate areolation and labiate processes. Scale bar = 10 µm. J. Internal view of valve mantle showing curved labiate processes and closer areolation. Scale = 2 µm. Fig. K. Internal oblique view of theca with wide valvocopula. Scale = 10 µm. Fig. L. External oblique view of broken end of valve, showing thick wall and tubular loculate areolae occluded by cribra at both surfaces. Scale = 1 µm. Fig. M. External oblique view of valvocopula with on fimbriate margin to the advalvar edge. Scale = 10 µm. (TIF) [file pone.0041890.s001.tif]

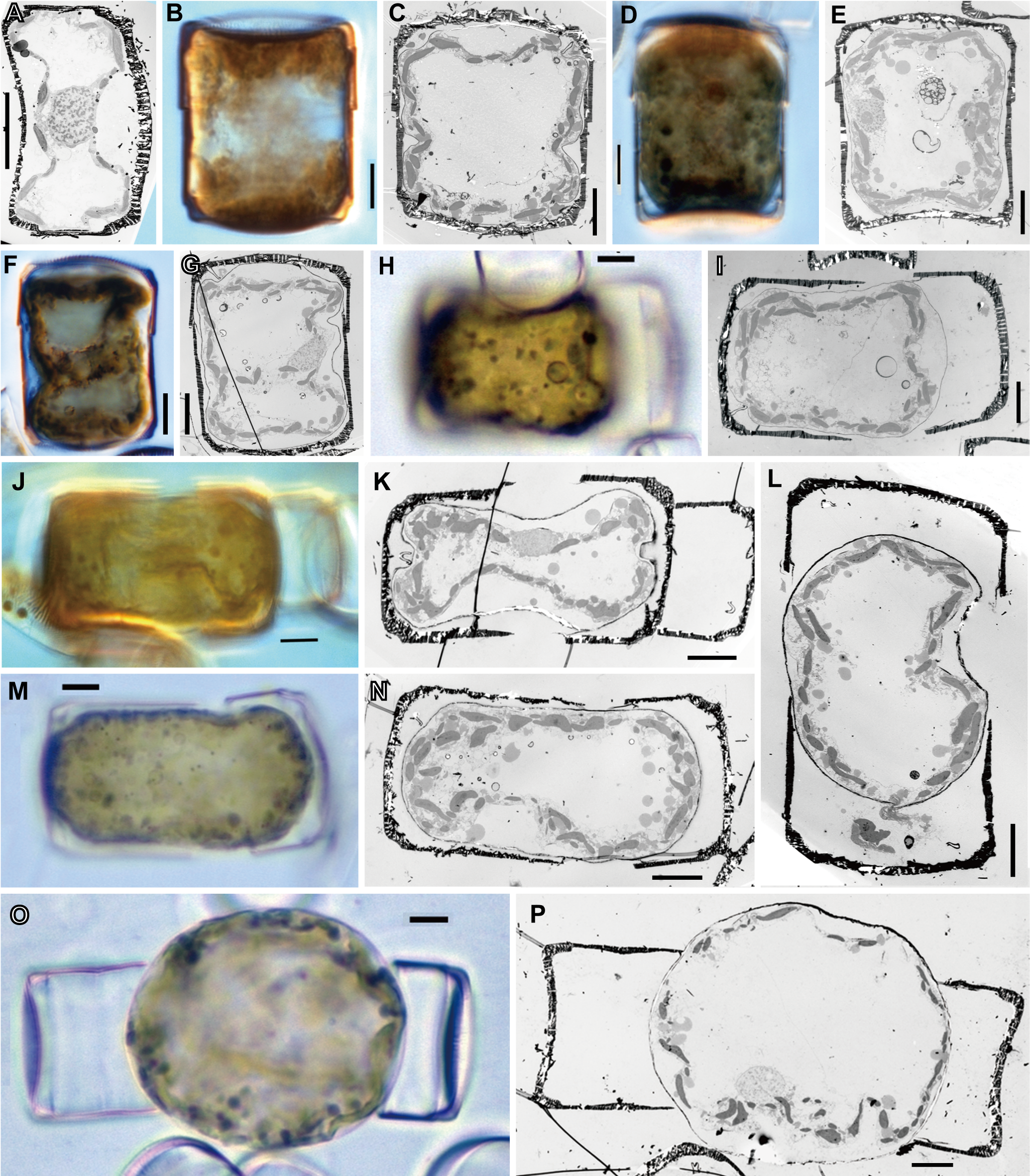

Supplement: Figure S2 — Cells selected for sectioning in Actinocyclus . A, C, E, G, I, K, L, N, P. Transmission electron micrographs. B, D, F, J, M, O. Light micrographs. B and C, D and E, F and G, H and I, J and K, M and N, O and P show the same cells respectively before and after thin sectioning. All scales = 10 µm. A. Vegetative cell with a central nucleus. B, C. Example 1 of a young oogonium with a thin layer of scales (visible as a thin dark line surrounding the protoplast (contrast S2-A), still fully enclosed by its frustule. D, E. Example 2 of a young oogonium with a thin layer of scales. F, G. Example 3 of a young oogonium with a thin layer of scales; in this cell, although the protoplast is still fully enclosed, the tilt of the epitheca (at top) relative to the hypotheca suggests that the oogonium was beginning to open to permit fertilization. H, I. Oogonium with slightly contracted cell after presumed fertilization. J, K. An expanded oogonium with a thin layer of scales. L. An ellipsoidal zygote/young auxospore with a thick layer of scales (contrast the thickness of the scale layer with e.g. S2-C, E, I or N), indicating new scale addition since presumed fertilization. M, N. A fully expanded oogonium after presumed fertilization, still with a thin layer of scales. O, P. An inflated, subspherical auxospore with a layer of scales. (TIF) [file pone.0041890.s002.tif]
